# The *de novo* synthesis of ubiquitin: identification of deubiquitinases acting on ubiquitin precursors

**DOI:** 10.1038/srep12836

**Published:** 2015-08-03

**Authors:** Cláudia P. Grou, Manuel P. Pinto, Andreia V. Mendes, Pedro Domingues, Jorge E. Azevedo

**Affiliations:** 1 Instituto de Investigação e Inovação em Saúde (i3S), Universidade do Porto, Portugal; 2 Organelle Biogenesis and Function Group, Instituto de Biologia Molecular e Celular (IBMC), Universidade do Porto, Rua do Campo Alegre 823, 4150-180 Porto, Portugal; 3 Mass Spectrometry Centre, UI-QOPNA, Department of Chemistry, University of Aveiro, 3810-193 Aveiro, Portugal; 4 Instituto de Ciências Biomédicas Abel Salazar (ICBAS), Universidade do Porto, Rua de Jorge Viterbo Ferreira 228, 4050-313 Porto, Portugal

## Abstract

Protein ubiquitination, a major post-translational modification in eukaryotes, requires an adequate pool of free ubiquitin. Cells maintain this pool by two pathways, both involving deubiquitinases (DUBs): recycling of ubiquitin from ubiquitin conjugates and processing of ubiquitin precursors synthesized *de novo*. Although many advances have been made in recent years regarding ubiquitin recycling, our knowledge on ubiquitin precursor processing is still limited, and questions such as when are these precursors processed and which DUBs are involved remain largely unanswered. Here we provide data suggesting that two of the four mammalian ubiquitin precursors, UBA52 and UBA80, are processed mostly post-translationally whereas the other two, UBB and UBC, probably undergo a combination of co- and post-translational processing. Using an unbiased biochemical approach we found that UCHL3, USP9X, USP7, USP5 and Otulin/Gumby/FAM105b are by far the most active DUBs acting on these precursors. The identification of these DUBs together with their properties suggests that each ubiquitin precursor can be processed in at least two different manners, explaining the robustness of the ubiquitin *de novo* synthesis pathway.

Protein ubiquitination is one of the most important post-translational modifications. It is used to control the half-life of proteins via the ubiquitin-proteasome system^1^ and also to regulate in a reversible manner a myriad of biological pathways such as DNA damage repair^2^, signal transduction^3^, and protein sorting^4^,^5^. The fine-tuned regulation of biological processes by ubiquitination relies, on one hand, on the action of the ubiquitin-conjugating cascade, a complex set of enzymes that conjugate ubiquitin to proteins^6^,^7^, and on the other hand, on deubiquitinases (DUBs), hydrolases that remove ubiquitin molecules from the modified proteins, thus ensuring the transient nature of ubiquitination.

DUBs are a diverse class of proteases comprising about 100 members in mammals (reviewed in ref. 8). In addition to removing ubiquitin from ubiquitinated proteins, DUBs are also involved in the *de novo* synthesis of ubiquitin. The participation of DUBs in this process is mandatory because ubiquitin is synthesized by cytosolic ribosomes in a precursor form that must be processed to yield active ubiquitin (reviewed in ref. 9). In mammals there are four such precursors, each encoded by a different gene^10^,^11^. UBA52 and UBA80 (hereafter referred to as Ub-RPs) comprise a single ubiquitin molecule C-terminally fused to a ribosomal protein, L40 and S27A, respectively^12^,^13^. UBB and UBC (polyUbs) are polymers of ubiquitins (3–4 and 7–10 ubiquitins, respectively, depending on the organism) linked in a “head-to-tail” fashion, followed by a variable C-terminal extension that goes from a single amino acid to a few dozens^14^.

The DUBs involved in ubiquitin precursors processing remain largely undefined^15^. Early studies, performed when just a few mammalian DUBs were known, implicated the ubiquitously expressed UCHL3 and the neuronal/gonad-specific UCHL1 in the processing of Ub-RPs^14^,^16^,^17^,^18^ whereas USP5 was implicated in polyUbs processing^19^. However, in the meantime, the number of known DUBs increased several fold and the *in vitro* characterization of their substrate specificities^20^,^21^,^22^ raises the possibility that many of them may also process ubiquitin precursors. The fact that mice lacking both UCHL3 and UCHL1 are viable and fertile^23^ supports this notion, at least in the case of Ub-RPs.

Another issue that remains unclear regards the timing of processing. Despite some earlier work on this topic^14^,^24^ (see Discussion), it is still unknown if processing of ubiquitin precursors is co-translational, post-translational or both. Clearly, additional work is needed to clarify these issues.

## Results

### Ubiquitin precursor processing: a co- or post-translational event?

Ubiquitin precursors are synthesized on cytosolic ribosomes and rapidly processed into mature ubiquitin by DUBs^25^ , but whether processing is co-translational, post-translational or both remains undefined. We reasoned that a rabbit reticulocyte lysate (RRL)-based translation system^26^, which is highly active in protein synthesis and is enriched in all the associated quality control machinery, including components of the ubiquitin-proteasome system^27^, and DUBs^28^, would replicate these events *in vitro* and provide insights into this question.

**Figure 1:**
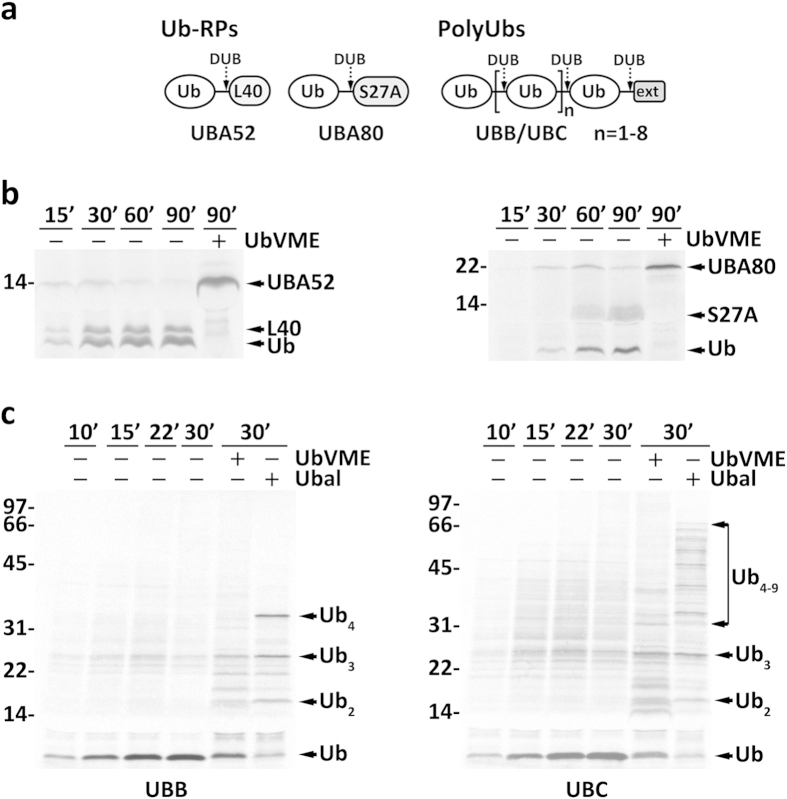
Synthesis and processing of ubiquitin precursors. (**a**) Schematic representation of the four mammalian ubiquitin precursors. UBA52 and UBA80 are ubiquitin-ribosomal protein fusions (Ub-RPs) in which a single ubiquitin molecule is linked to a ribosomal protein, L40 and S27A, respectively. UBB and UBC are ubiquitin polymers (polyUbs) of different lengths followed by a C-terminal extension (ext) that ranges from a single amino acid (e.g., a cysteine and a tyrosine in human and mouse UBB, respectively) to a small polypeptide (e.g., 50 amino acid residues in mouse UBC). The number of ubiquitins in mouse UBB and UBC is 4 and 9; human UBB and UBC have 3 and 9 ubiquitins, respectively. Generation of free ubiquitin from these precursors requires the action of deubiquitinases (DUB; dashed arrows). (**b**) ^35^S-labeled mouse Ub-RPs UBA52 and UBA80 and (**c**) mouse polyUbs UBB and UBC were synthesized *in vitro* in the absence or presence of DUB inhibitors (HA-UbVME or HA-Ubal), as indicated. Samples were withdrawn at the indicated time points. UBA52, UBA80, its corresponding processed products (L40, S27A and Ub), and partially processed UBB and UBC species are indicated. Note that S27A co-migrates with the abundant hemoglobin from the reticulocyte lysate and therefore is barely visible at earlier time points. In (**b**,**c**) numbers to the left indicate the molecular weights of protein standards in kDa.

We first programmed the RRL with cDNAs encoding the four mouse ubiquitin precursors (see Fig. 1a for a schematic representation of these molecules), and asked whether we could detect the corresponding precursor forms. As shown in Fig. 1b, although a major fraction of both Ub-RPs was cleaved in these reactions, unprocessed forms were easily detectable. Apparently, at least a fraction of Ub-RPs reaches the end of translation still intact. Processing of Ub-RPs was blocked when HA-tagged ubiquitin-vinylmethyl ester (HA-UbVME), an irreversible inhibitor of many DUBs belonging to the cysteine protease family^29^, was added to the reactions.

Different results were obtained for polyUbs. In both cases no intact precursors were detected at any time point analyzed indicating that processing is a fast event (Fig. 1c). Interestingly, HA-UbVME blocked processing of polyUbs only partially, whereas a much stronger inhibition, almost complete in the case of UBB, was observed in the presence of HA-tagged ubiquitin aldehyde (HA-Ubal), another inhibitor that specifically targets DUBs of the cysteine protease family^30^.

**Figure 2:**
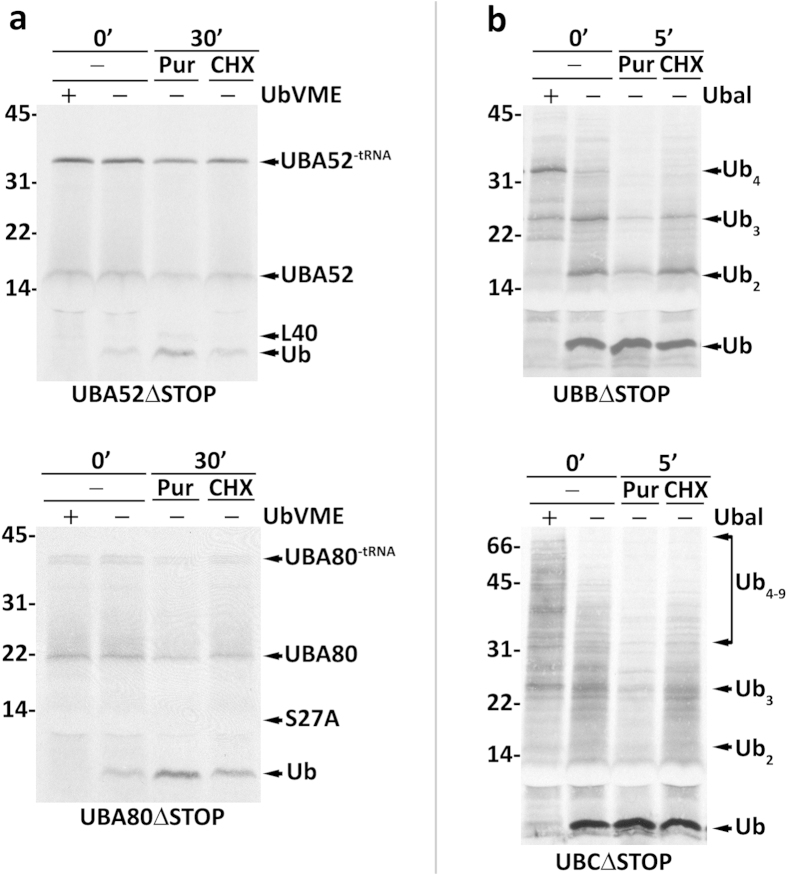
Accessibility of ribosome-stalled ubiquitin precursors to DUBs. Mouse ubiquitin precursors were *in vitro* synthesized using stopless mRNAs (ΔSTOP). Synthesis was for (**a**) 30 min or (**b**) 45 min, in the absence or presence of HA-UbVME or HA-Ubal, as indicated. Aliquots of synthesis reactions performed in the absence of inhibitor were further incubated in the presence of either puromycin (Pur) or cycloheximide (CHX) for the indicated time periods. In (**a**) the peptidyl-tRNA species (UBA52^-tRNA^ and UBA80^-tRNA^), unprocessed UBA52 and UBA80, as well as the corresponding processing products, L40, S27A and ubiquitin (Ub), are indicated. Note that S27A co-migrates with the abundant hemoglobin from the reticulocyte lysate hampering its detection. In (**b**) partially processed UBB and UBC species are indicated. Numbers to the left indicate the molecular weights of protein standards in kDa.

Aiming at better understanding how newly synthesized ubiquitin precursors are processed we asked whether ubiquitin precursors artificially arrested at the ribosome are accessible to DUBs. For this we explored the fact that translation of an mRNA lacking a stop codon proceeds normally until its last codon; at this point, however, the ribosome nascent chain complex is not recognized by a releasing factor and thus the nascent polypeptide remains in the ribosome as a peptidyl-tRNA species^31^. The peptidyl moiety of this species has its last C-terminal residue covalently linked by an ester bond to the tRNA present in the ribosomal P-site, its last 30–40 C-terminal amino acid residues buried in the ribosomal polypeptide exit tunnel, and the remaining N-terminal residues outside the ribosome^32^. Note that the ester bonds of different peptidyl-tRNAs display different susceptibilities to hydrolysis during SDS-PAGE (see ref. 33). Thus, some peptidyl-tRNAs species are quite resistant and thus easily detectable, whereas others undergo massive hydrolysis during electrophoresis yielding essentially the free peptidyl moiety (see ref. 34). As shown in Fig. 2a, the vast majority of UBA52 and UBA80 produced by translation of stopless mRNAs were detected as a mixture of peptidyl-tRNAs and peptidyl moieties even in the absence of HA-UbVME (note the smear between the two species, the result of hydrolysis of the peptidyl-tRNA during electrophoresis; see ref. 35). Importantly, only a small amount of free ubiquitin was detected under these conditions. This ubiquitin could result from residual co-translational processing of ribosomal peptidyl-tRNAs and/or from processing of abortive termination products which are common in the RRL^31^. The latter possibility probably predominates for UBA52 because further incubation of the lysate in the presence of cycloheximide (to stop translation) for 30 min did not increase free ubiquitin. For UBA80 a slight increase of ubiquitin was detected upon this incubation, suggesting that UBA80 is partially accessible to DUBs even when still attached to a ribosome. Notably, however, a large increase of ubiquitin was observed in both cases when the 30-min incubation was performed in the presence of puromycin, a tRNA-mimetic antibiotic that triggers release of the peptidyl moiety from the ribosome^36^. Clearly, Ub-RPs arrested at ribosomes are, at best, very poor DUB substrates. This finding together with the data in Fig. 1b strongly suggest that Ub-RPs are processed mostly post-translationally.

The results obtained with UBB and UBC are shown in Fig. 2b. In both cases the majority of the precursor forms were no longer detected, except when HA-Ubal was present during synthesis. Thus, in contrast to Ub-RPs, polyUbs arrested at the ribosome are quite accessible to HA-Ubal-sensitive DUBs. However, despite the fact that these species were maintained at the ribosome for many more minutes than they normally would, incomplete processed forms were still abundant. This is particularly visible for UBB, where species containing 2, 3 and even 4 ubiquitin moieties can be detected (Fig. 2b, upper panel). Note that all these species represent ribosome-associated peptidyl-tRNAs because further incubation in the presence of puromycin (but not cycloheximide) resulted in their disappearance with a concomitant increase of ubiquitin. We conclude that although both UBB and UBC are clearly accessible to DUBs during translation, a fraction of both ubiquitin precursors probably escapes co-translational cleavage in a partially processed form. In the case of UBB it is even possible that some intact precursor is released from the ribosome.

### Identification of mouse liver DUBs acting on ubiquitin precursors

The results above suggest that many of the processing events necessary to convert ubiquitin precursors into ubiquitin occur post-translationally. As a first step to identify the DUBs involved in these events we produced recombinant versions of mouse ubiquitin precursors and tested them in enzymatic assays with a mouse liver cytosolic fraction. As shown in Supplementary Fig. S1, all four precursors are efficiently processed by this fraction. Interestingly, and similarly to the results described above, we found that processing of Ub-RPs was completely blocked by HA-UbVME, whereas that of polyUbs was almost completely blocked only by HA-Ubal.

**Figure 3:**
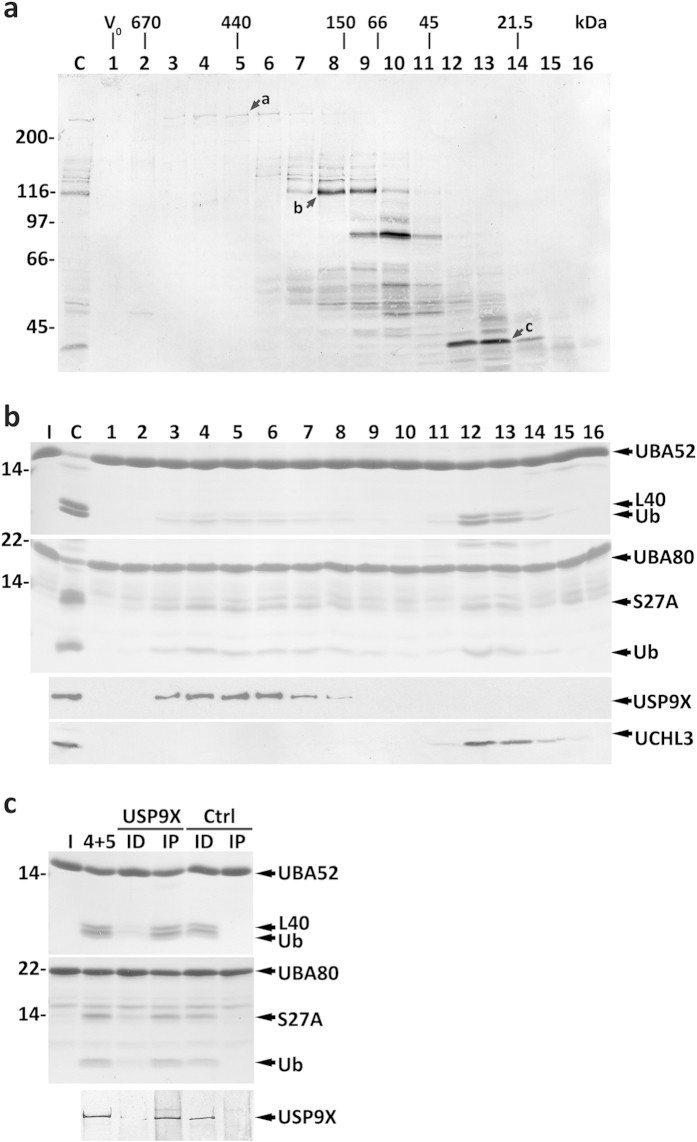
Characterization of mouse liver cytosolic DUBs acting on Ub-RPs. (**a**) SEC profile of HA-UbVME-reactive DUBs from mouse liver cytosol. Total cytosolic proteins (lane C) and the corresponding SEC fractions (lanes 1–16) were reacted with HA-UbVME and analyzed by SDS-PAGE/western blotting with an anti-HA antibody. Arrows a–c indicate HA-UbVME-reactive DUBs that co-elute with enzyme activities described in the main text. The elution positions of molecular mass protein standards, as well as the void volume (V_0_), are indicated. (**b**) SDS-PAGE/Coomassie blue staining analyses of UBA52 and UBA80 processing by SEC fractions (first and second panels, respectively). Lane C, total cytosolic proteins were also assayed. Ubiquitin (Ub) and the ribosomal proteins L40 and S27A are indicated. The elution profiles of USP9X and UCHL3 are also shown (lower panels). (**c**) Pooled fractions 4 and 5 from SEC were subjected to an immunoprecipitation/immunodepletion assay using control (lanes Ctrl) or anti-USP9X IgGs (lanes USP9X). Pooled fractions (4+5) and the corresponding immunoprecipitated (lanes IP) and immunodepleted fractions (lanes ID) were assayed for UBA52 and UBA80 cleavage (upper and middle panels, respectively). The distribution of USP9X in the samples is also shown (lower panel). Lanes I, recombinant Ub-RP fusion proteins used in the assays. Numbers to the left indicate the molecular weights of protein standards in kDa.

We next subjected mouse cytosolic proteins to size-exclusion chromatography (SEC), and tested the fractions in activity assays. Fraction aliquots were also incubated with HA-UbVME and subjected to western blotting using an anti-HA antibody (see Fig. 3a). The aim was to correlate the activity profiles with specific HA-UbVME-reactive DUBs, thus narrowing down the number of candidates in subsequent identification and validation experiments.

As shown in Fig. 3b, two activity peaks were detected with Ub-RPs: one in fractions 12–13, in the 30-kDa region of the SEC, and a broader one centered in the 440-kDa region (fractions 3–8). Alignment of the activity profiles with the western blot of Fig. 3a, revealed two HA-UbVME-reactive proteins (bands a and c) co-eluting with the activity peaks. A comparison of the HA-UbVME labeling pattern obtained for mouse liver cytosolic proteins (Fig. 3a) with patterns described previously (see also Supplementary Fig. S2), together with available SEC data^29^,^37^,^38^,^39^,^40^,^41^, suggested that the large molecular mass DUB might be USP9X, whereas the smaller one could be UCHL3. The capacity of UCHL3 to process Ub-RPs is documented^14^. Western blot analyses of SEC fractions using anti-USP9X and anti-UCHL3 antibodies are compatible with this possibility (Fig. 3b, third and fourth panels, respectively).

To determine whether USP9X and UCHL3 are the DUBs of interest we performed immunoprecipitation/immunodepletion experiments and tested the fractions obtained in activity assays. The results obtained with anti-USP9X IgGs (Fig. 3c) show that the Ub-RPs-cleaving activity parallels the distribution of USP9X, strongly suggesting that USP9X is indeed the major activity eluting in the 440-kDa region of the SEC. Attempts to perform similar experiments for UCHL3 were not successful because none of the antibodies tested was able to immunoprecipitate the enzyme (but see below).

**Figure 4:**
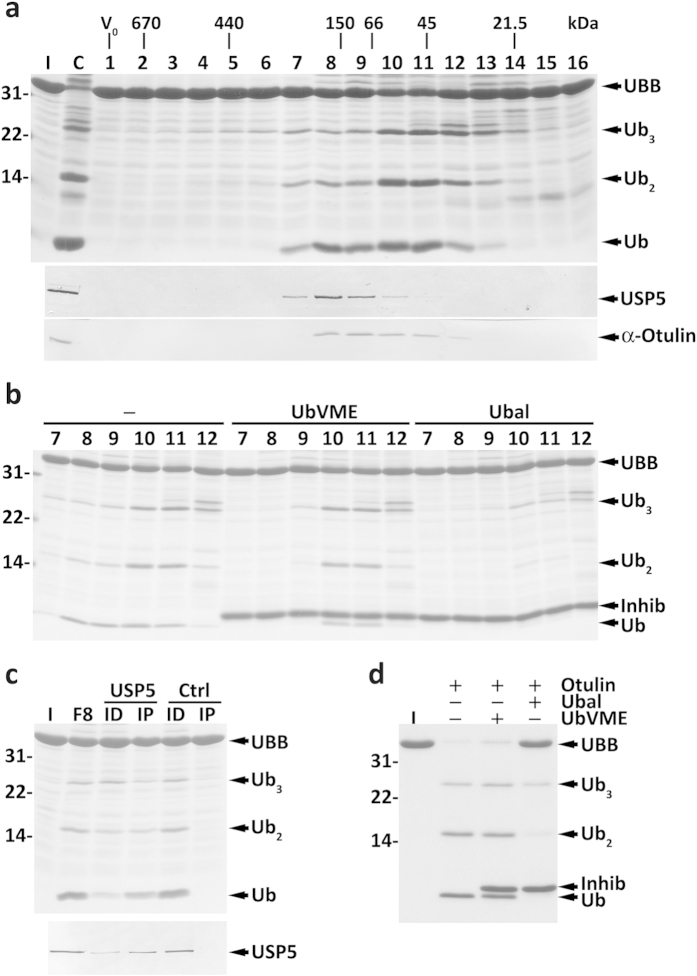
Characterization of mouse liver cytosolic DUBs acting on polyUbs. (**a**) SDS-PAGE/Coomassie blue staining analysis of UBB processing by SEC fractions (upper panel). Lane C, total cytosolic proteins were also assayed. The elution profiles of USP5 (middle panel) and of the protein recognized by the anti-Otulin antibody (lower panel) are also shown. The elution positions of molecular mass protein standards, as well as the void volume (V_0_), are indicated. (**b**) The UBB cleavage activity comprises an HA-UbVME-sensitive and an HA-UbVME-insensitive component. SEC fractions 7–12 were preincubated in the absence or presence of HA-UbVME or HA-Ubal and used in UBB processing activity assays. Inhib, HA-UbVME or HA-Ubal. (**c**) SEC fraction 8 was subjected to an immunoprecipitation/immunodepletion assay using control (lanes Ctrl) or anti-USP5 IgGs (lanes USP5). Fraction 8 (F8) and the corresponding immunoprecipitated (lanes IP) and immunodepleted fractions (lanes ID) were assayed for processing activity (upper panel). The distribution of USP5 in the samples is shown (lower panel). (**d**) Recombinant Otulin (10 ng) pretreated or not with HA-UbVME or HA-Ubal, as indicated, was incubated with UBB for 10 min at 37 °C. A Coomassie blue-stained gel is shown. Cleavage intermediates (Ub_3_ and Ub_2_) and ubiquitin (Ub) are indicated. Lane I, recombinant mouse UBB used in the assays. Numbers to the left indicate the molecular weights of protein standards in kDa.

The activity profile obtained with UBB is shown in Fig. 4a. A single but rather broad activity peak centred in fractions 8–11 was detected. Interestingly, although similar amounts of ubiquitin were detected in those fractions, the amounts of uncleaved and partially cleaved UBB in fractions 8–9 differ from those in fractions 10–11. This finding suggested that there are at least two different DUBs cleaving UBB. Data supporting this interpretation were obtained when fractions 7–12 were treated with HA-UbVME or HA-Ubal and used in activity assays. As shown in Fig. 4b, the activity in fractions 8–9 is sensitive to both HA-UbVME and HA-Ubal, whereas that in fractions 10–11 is largely inhibited only by HA-Ubal. The same results were obtained with UBC (see Supplementary Fig. S3a). Note that pretreatment of polyUbs with UCHL3 to remove their C-terminal extensions (see below) does not change the SEC activity profiles (see Supplementary Fig. S3b, and data not shown). This suggests that no DUB(s) displaying a large activity toward polyUbs lacking C-terminal extensions, but unable to process polyUbs having them, were missed in these assays.

The blot in Fig. 3a shows an abundant HA-UbVME-reactive protein in fractions 8–9 (arrow b) that might correspond to the HA-UbVME-sensitive DUB that cleaves polyUbs. We compared our HA-UbVME labeling and SEC results (Fig. 3a) with data described by other groups^19^,^29^,^37^,^38^,^40^ (see also Supplementary Fig. S2) and reasoned that this DUB might be USP5, an enzyme previously implicated in polyUbs processing^19^. Accordingly, western blot analyses show that the enzyme co-elutes with the polyUbs processing activity (Fig. 4a, middle panel). To test this possibility, SEC fraction 8 was subjected to immunodepletion/immunoprecipitation experiments using the anti-USP5 antibody. As shown in Fig. 4c, the processing activity paralleled the amounts of USP5 in each fraction. Thus, USP5 is the main HA-UbVME-sensitive mouse liver DUB acting on polyUbs.

The fact that the DUB in fractions 10–11 is HA-UbVME-insensitive complicated its identification. Efforts to isolate it by affinity chromatography using HA-Ubal were not successful. Therefore, we searched the literature for DUBs of the cysteine protease family possessing a molecular mass of 40–60 kDa that are not promptly inhibited by HA-UbVME or similar probes (e.g., ubiquitin-vinyl sulfone). We found only three DUBs with these properties: Ataxin-3^40^,^42^, Otub1^22^,^29^,^43^ and Otulin^22^,^44^,^45^. Otub1 does not cleave linear ubiquitin chains^22^,^43^, whereas Ataxin-3 elutes upon SEC as a 150-kDa protein (ref. 46, and data not shown). Otulin, in contrast, cleaves linear ubiquitin chains quite efficiently^22^,^44^,^45^. Importantly, despite its insensitivity to UbVME, we found that Otulin is largely inhibited by Ubal (Fig. 4d), similarly to the DUB in fractions 10–11 of the SEC (Fig. 4b). The reason why Otulin presents this behaviour is unknown, but we note that divergent sensitivities of a given DUB to different ubiquitin-based probes is not an uncommon phenomenon^22^,^29^.

To test the hypothesis that Otulin is the DUB of interest we analysed its elution profile upon SEC. Unfortunately, the results were not clear - although a portion of the mouse protein recognized by this antibody elutes in fractions 10–11, the majority elutes in fractions 8–9 (Fig. 4a, third panel). Attempts to immunoprecipitate this mouse protein with anti-Otulin antibodies were unsuccessful, thus hampering us from confirming whether this protein is really Otulin. Nevertheless, as shown below, Otulin is the major HA-UbVME-insensitive DUB acting on polyUbs.

### HeLa cell DUBs acting on ubiquitin precursors

To determine whether the results obtained for mouse liver are valid for human cells and, if so, to test the involvement of Otulin and UCHL3 in ubiquitin precursor processing, we repeated some of the experiments using HeLa cells.

**Figure 5:**
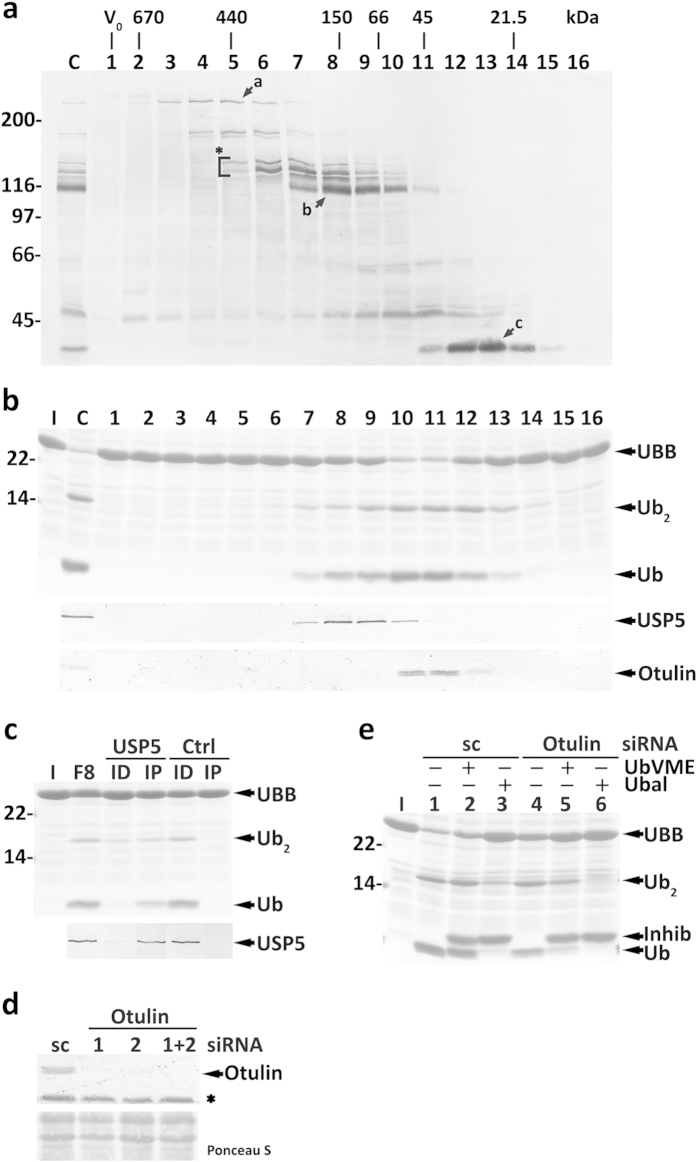
Characterization of HeLa cells cytosolic DUBs acting on human UBB. (**a**) Total cytosolic proteins (lane C) and the corresponding SEC fractions (lanes 1–16) were reacted with HA-UbVME and analyzed by SDS-PAGE/western blot with an anti-HA antibody. The asterisk and arrows a-c indicate HA-UbVME-reactive DUBs that co-elute with enzyme activities described in the main text. The elution positions of molecular mass protein standards, as well as the void volume (V_0_), are indicated. (**b**) SDS-PAGE/Coomassie blue staining analysis of UBB processing by SEC fractions (upper panel). Lane C, total cytosolic proteins were also assayed. The elution profiles of USP5 (middle panel) and Otulin (lower panel) are also shown. (**c**) Fraction 8 from SEC was subjected to an immunoprecipitation/immunodepletion assay using control (lanes Ctrl) or anti-USP5 IgGs (lanes USP5). Fraction 8 (F8) and the corresponding immunoprecipitated (lanes IP) and immunodepleted fractions (lanes ID) were assayed for UBB processing activity (upper panel). The distribution of USP5 in the samples is shown (lower panel). (**d**) Otulin knockdown in HeLa cells. Western blot analysis of Otulin in HeLa cells transfected with Otulin-specific siRNA oligos #1 and/or #2, or with a scrambled (sc) control. The asterisk indicates a protein recognized by the anti-Otulin antibody in total homogenates, but not in cytosolic fractions. The corresponding Ponceau S-stained membrane is shown to assess protein loadings. (**e**) *OTULIN* knocked-down HeLa cell total extracts have decreased HA-UbVME-insensitive UBB processing activity. Inhib, HA-UbVME or HA-Ubal. In (**b**,**c**,**e**) the cleavage intermediate (Ub_2_), and ubiquitin (Ub) are indicated. Lanes I, recombinant UBB used in the assays. In (**a**–**c**,**e**) numbers to the left indicate the molecular weights of protein standards in kDa.

The SEC activity profiles of HeLa cytosolic proteins using HA-UbVME and human UBB are shown in Fig. 5a,b, respectively. Similarly to the results above (Fig. 4a), a broad activity peak was detected with UBB (Fig. 5b, fractions 8–12). Importantly, this peak also comprises two components, one sensitive and the other insensitive to HA-UbVME (Supplementary Fig. S4a, fractions 8–9 and 10–11, respectively). The HA-UbVME-sensitive activity co-elutes with USP5 (Fig. 5b, middle panel) and, indeed, immunoprecipitation/immunodepletion experiments confirmed that USP5 is the main UBB-processing DUB in these fractions (Fig. 5c).

In contrast to the results obtained with mouse liver, the anti-Otulin antibody recognized a ~43-kDa double band that co-elutes with the HA-UbVME-insensitive activity (Fig. 5b, lower panel, fractions 10–11). The reason for the two bands is unknown, but they do correspond to Otulin, as determined by siRNA knockdown experiments (Fig. 5d). None of the anti-Otulin antibodies tested immunoprecipitates the enzyme. Thus, total extracts from control and *OTULIN* knocked-down HeLa cells were prepared and assayed for UBB-processing activity. As shown in Fig. 5e, extracts from the *OTULIN* knocked-down cells present almost no HA-UbVME-insensitive activity (compare lanes 2 and 5, respectively). Together, these results strongly suggest that USP5 and Otulin are by far the most active DUBs acting on polyUbs.

**Figure 6:**
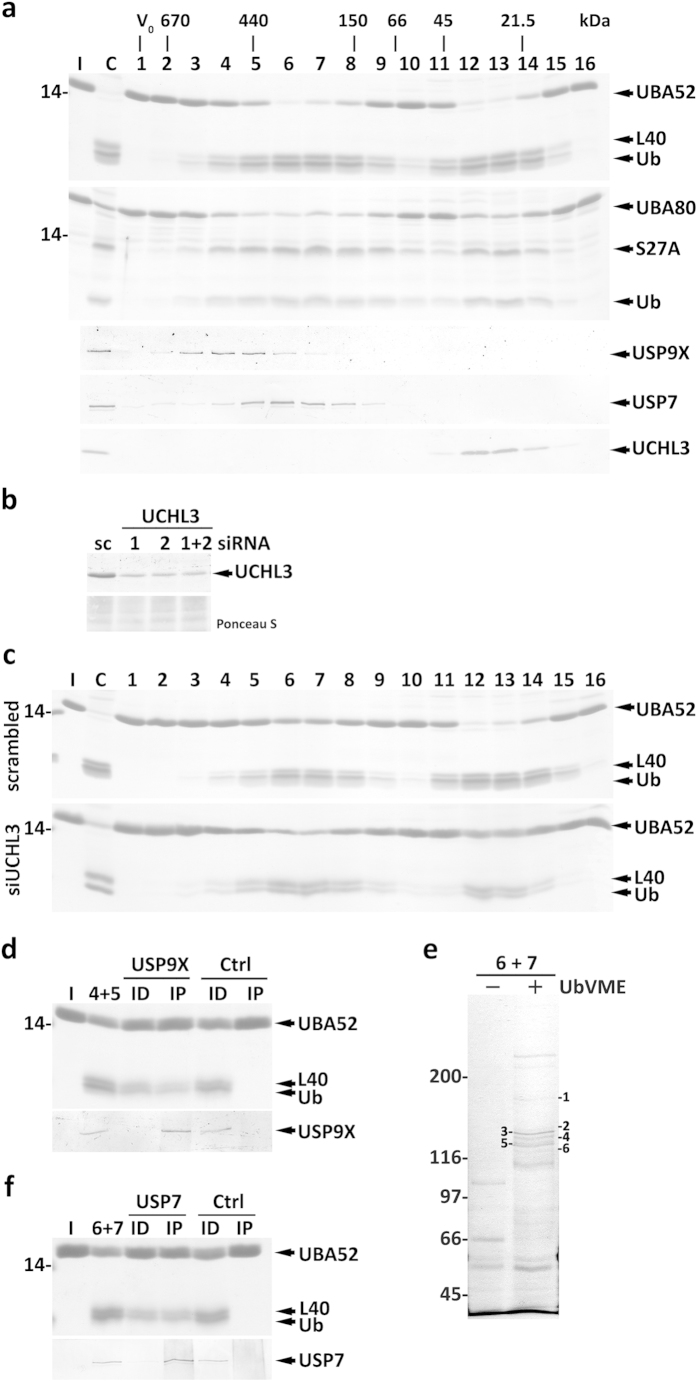
Characterization of HeLa cells cytosolic DUBs acting on Ub-RPs. (**a**) SDS-PAGE/Coomassie blue staining analyses of UBA52 and UBA80 processing by HeLa cells cytosolic SEC fractions (first and second panels, respectively). Lane C, total cytosolic proteins were also assayed. Ubiquitin (Ub) and the ribosomal proteins L40 and S27A are indicated. The elution profiles of USP9X, USP7 and UCHL3 are also shown. The protein standards used to calibrate the column, as well as the void volume (V_0_), are indicated. (**b**) Western blot analysis of UCHL3 in HeLa cells transfected with UCHL3-specific siRNA oligos #1 and/or #2, or with a scrambled (sc) control. The corresponding Ponceau S-stained membrane is shown to assess protein loadings. (**c**) SEC profiles of UBA52-cleavage activity in control (scrambled) and *UCHL3* knocked-down (siUCHL3) HeLa cells cytosol. (**d**) Pooled SEC fractions 4 and 5 were subjected to an immunoprecipitation/immunodepletion assay using control (lanes Ctrl) or anti-USP9X IgGs (lanes USP9X). Pooled fractions (4 + 5) and the corresponding immunoprecipitated (lanes IP) and immunodepleted fractions (lanes ID) were assayed for UBA52 cleavage (upper panel). The distribution of USP9X in the samples is shown (lower panel). (**e**) Pooled fractions 6 and 7 (6 + 7) from SEC were incubated with HA-UbVME (lane +) or with HA-Ub (lane -) and subjected to immunoprecipitation using anti-HA-conjugated beads. A Coomassie blue-stained gel is shown. Numbers indicate protein bands of interest that were subjected to mass spectrometry (see Supplementary Table S1). (**f**) Immunoprecipitation/immunodepletion assay exactly as described in (**d**) but using pooled SEC fractions 6 and 7, and anti-USP7 IgGs. Lanes I in (**a**,**c**,**d**,**f**), recombinant UBA52 or UBA80, as indicated. In (**a**,**c**–**e**) numbers to the left indicate the molecular weights of protein standards in kDa.

The SEC activity profiles of HeLa cytosolic proteins using Ub-RPs as substrates are shown in Fig. 6a. Two peaks were detected, one in fractions 12–13 and the other in fractions 5–8. The activity in fractions 12–13 co-elutes with UCHL3 (Fig. 6a, lower panel), as found for mouse liver (see Fig. 3b). To test the contribution of UCHL3 to this activity, a cytosolic fraction from *UCHL3* knocked-down HeLa cells (see Fig. 6b) was subjected to SEC, and the fractions were assayed for Ub-RPs-processing activity. As shown in Fig. 6c and Supplementary Fig. S4b, the activity detected in fractions 12–13 was now much lower. Thus, UCHL3 is a major Ub-RPs-processing enzyme in HeLa cells.

Unexpectedly, the activity peak in fractions 5–8 does not parallel USP9X distribution (Fig. 6a, third panel from top). Instead, the Ub-RP-cleaving activity co-elutes with a group of HA-UbVME-reactive proteins displaying a molecular mass of about 120 kDa upon SDS-PAGE (see asterisk in Fig. 5a). This finding does not mean that USP9X from HeLa cells is unable to process Ub-RPs. As shown in Fig. 6d and Supplementary Fig. S4c, immunoprecipitated USP9X does cleave Ub-RPs. Rather, it is apparent that HeLa cells possess another, far more active, DUB with this capacity. To identify this enzyme, SEC fractions 6–7 were pooled, incubated with HA-UbVME, and purified using anti-HA beads. Several DUBs were recovered (see Fig. 6e) and identified by mass spectrometry (Supplementary Table S1). Western blot analyses of SEC fractions using antibodies directed to these DUBs (i.e., USP4, USP7, USP8, USP15, USP47 and USP48; Supplementary Fig. S4d) revealed USP7 as the one that elutes more closely with the second activity peak (Fig. 6a, fourth panel from top). Note that although USP7 has been reported to be more concentrated in the nucleus, significant amounts of the enzyme are also present in the cytosol^47^,^48^. Importantly, immunodepletion/immunoprecipitation experiments performed with SEC fractions 6–7 suggest that about half of the Ub-RPs-processing activity they contain is due to USP7 (Fig. 6f and Supplementary Fig. S4e). A fraction of the activity remaining in the immunodepleted fraction is probably due to USP9X, which is also present in these SEC fractions (see Fig. 6a, third panel from top). However, it seems that other DUB(s) contributes to the observed activity. Attempts to identify it using immunoprecipitation experiments were either not possible, because the antibodies did not work in this type of experiments, or yielded negative results, as it was the case for USP15 and USP48 (data not shown). Regardless, it is clear that USP7 is a major Ub-RP-processing enzyme in these SEC fractions.

### USP5 efficiently removes C-terminal extensions from polyUbs

**Figure 7:**
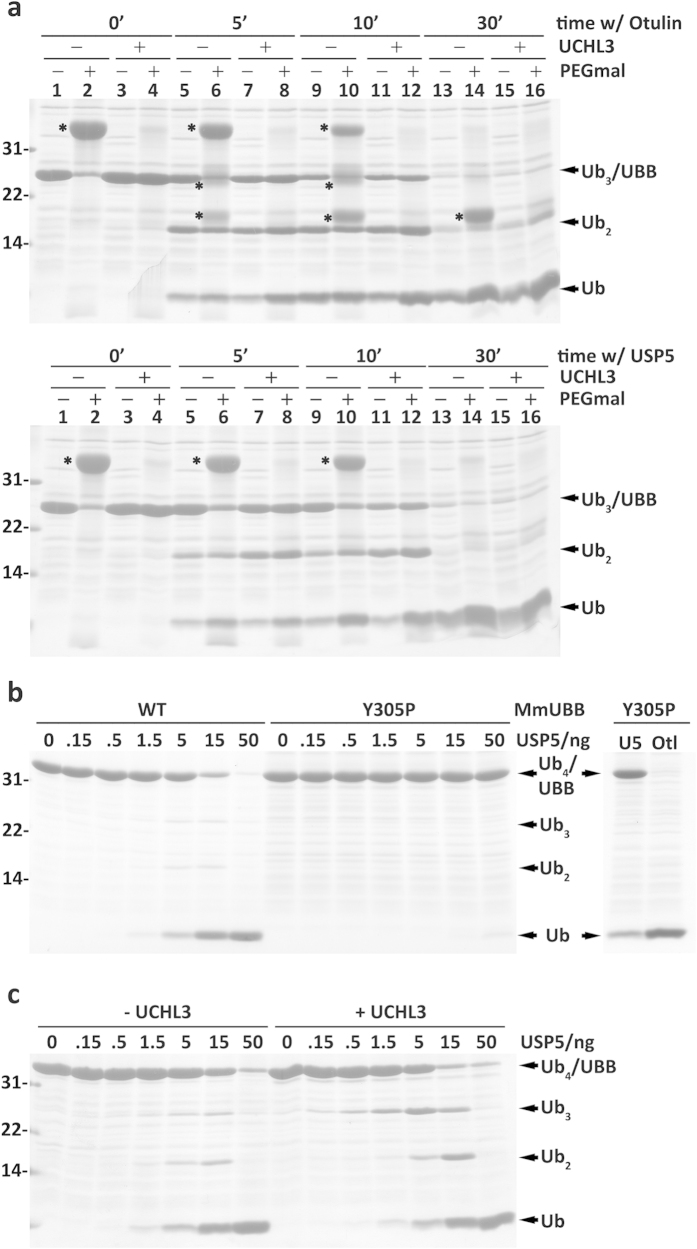
USP5 removes the C-terminal extension of UBB. (**a**) Human UBB pretreated or not with UCHL3 was incubated with either Otulin (8 ng/lane; upper panel) or USP5 (40 ng/lane; lower panel) for the indicated periods of time. Samples were then subjected to pegylation (+PEGmal), as indicated. Pegylated species (UBB and cleavage products) are marked with asterisks. Note that in the Otulin assay, Ub_2_ and Ub also indicate species containing the C-terminal cysteine. (**b**) Mouse UBB (WT) and UBBY305P (Y305P) were incubated with increasing amounts of USP5 for 30 min at 37 °C (left panel). Processing of UBBY305P with 1 μg of USP5 (lane U5) or 10 ng of Otulin (lane Otl) is also shown (right panel). (**c**) Mouse UBB pretreated or not with UCHL3, as indicated, was incubated at 37 °C for 30 min with increasing amounts of USP5, as specified. Coomassie blue-stained gels are shown. Numbers to the left indicate the molecular weights of protein standards in kDa.

USP5 acts as an exo-(iso)amidase releasing ubiquitin moieties from the proximal end of unanchored ubiquitin chains in a non-processive manner^49^. The enzyme has several ubiquitin-binding domains one of which, the S1’ site, interacts with the C-terminally located ubiquitin, i.e., the proximal ubiquitin^49^,^50^,^51^. It is known that occupancy of the S1’ site by the proximal ubiquitin activates USP5 and that the C-terminal glycine (G76) of this ubiquitin is determinant for this activation^52^,^53^. Indeed, deletion of G76 or its esterification reduces USP5 activity^49^. None of the polyUbs used here possesses a free G76 at their proximal ubiquitin (Supplementary Fig. S3b and S5, and see below). Yet, they are all efficiently processed. This finding could suggest that either USP5 S1’ site is structurally tolerant, accepting proximal ubiquitins with C-terminal extensions (mechanism #1), or that the first binding/hydrolysis event does not involve at all the S1’ site (mechanism #2). In the latter situation the proximal ubiquitin would occupy the catalytic domain of USP5 (the S1 site) and lose its carboxyl extension. Mechanism #1 predicts that the first monoubiquitin generated during processing by USP5 still has its C-terminal extension, whereas in mechanism #2 no such species is generated. To clarify this, a kinetic analysis of human UBB processing by USP5 and Otulin was performed. Human UBB was selected because G76 of its proximal ubiquitin is followed by a cysteine, the presence of which can be readily assessed by reaction with PEG-maleimide, a cysteine-specific pegylation reagent that increases the molecular mass of the modified proteins by approximately 10 kDa. UBB pretreated with recombinant UCHL3 was also included in these assays as a control. UCHL3 removes small extensions from the C terminus of (poly)ubiquitin but is not able to hydrolyze the peptide bond linking two ubiquitins^14^. As shown in Fig. 7a upper panel, processing of human UBB by Otulin yields mono- and di-ubiquitins still possessing the cysteine residue (lanes 6, 10 and 14). Otulin cleaves randomly because about 50% of the di-ubiquitins detected lack a cysteine (compare lanes 5 and 6). In contrast, no cysteine-containing mono- or di-ubiquitins were detected with USP5 (Fig. 7a, lower panel; see also Supplementary Fig. S6), a finding strongly favouring mechanism #2. Further data supporting this mechanism were obtained when a mouse mutant UBB possessing a proline instead of tyrosine at the C terminus (UBBY305P) was used in these assays. Cleavage of the ubiquitin-proline peptide bond by most DUBs is an inefficient process^14^,^54^. If USP5 cleaves polyUbs using mechanism #1, no difference in cleaving efficiency should be detected with UBBY305P, whereas processing through mechanism #2 should occur with slower kinetics. As shown in Fig. 7b, USP5 barely cleaves UBBY305P. Finally, USP5 cleaves UBB lacking the C-terminal tyrosine with only a slightly better efficiency than intact UBB (Fig. 7c). Collectively, these results show that USP5 efficiently removes the C-terminal extensions from polyUbs, a step that precedes hydrolysis of inter-ubiquitin peptide bonds.

## Discussion

Although ubiquitin was discovered 40 years ago, the mechanism of its *de novo* synthesis has not received much attention. Earlier studies led to the idea that ubiquitin precursors might be processed co-translationally. The evidence supporting this idea was of two types. First, it was found that ubiquitin precursors are much better processed when co-expressed in *Escherichia coli* with some DUBs than when incubated with the same enzymes *in vitro*^14^,^24^. Second, it has been shown that processing of engineered ubiquitin-fusion proteins by cytosolic DUBs is so fast *in vivo* that can even occur co-translationally, at least for large proteins^55^. Here we provide data strongly suggesting that this idea is only partially correct. Indeed, we found that Ub-RPs artificially arrested at the ribosome are, at best, very poor substrates for the DUBs present in the reticulocyte lysate. In contrast, when these precursor proteins were allowed to leave the ribosome, their processing was far more efficient. We did find clear evidence in favor of co-translational processing when characterizing polyUbs. However, even in these cases partially processed intermediates (and even traces of intact UBB) were still detected in the experiments with the stopless mRNAs (Fig. 2b). Collectively, these data suggest that Ub-RPs are mostly processed post-translationally whereas processing of polyUbs probably occurs through a combination of co- and post-translational mechanisms.

We next focused on the identities of the DUBs that process ubiquitin precursors. The biochemical approach used for this purpose aimed at identifying enzymes acting at the post-translational level. However, as discussed below, it is likely that one of these enzymes also plays a role during co-translational processing of polyUbs.

One of the DUBs found is UCHL3, an enzyme that was proposed to play a role in Ub-RPs processing many years ago^14^. Its identification in our assays re-enforces the validity of our approach and extends to both HeLa cells and mouse liver the idea that UCHL3 is one of the major enzymes involved in processing Ub-RPs. Importantly, our work also suggests that UCHL3 is not alone in this task. Indeed, USP9X (in mouse liver) and USP7 (in Hela cells) are also highly active in Ub-RPs processing. Actually, processing of mouse UBA80 seems to be even more efficient with USP9X than with UCHL3 (see Fig. 3b).

Similarly to the results on Ub-RPs, two DUBs highly active towards polyUbs were identified in this work. One of these enzymes, USP5, has been previously proposed to process polyUbs^19^. The results presented here completely support this idea. Interestingly, our results also suggest that USP5 can cleave efficiently polyUbs carrying a C-terminal extension but only when that extension fits into the S1’ site of the enzyme. Given the data in Fig. 7 showing that a proximal ubiquitin possessing a single proline at its C terminus is already too large to fit into the S1’ site of USP5, it is reasonable to assume that a ribosome will not fit either. This excludes USP5 from participating in any co-translational processing events, thus leaving a role for USP5 only in processing polyUbs, or fragments of them, that escaped co-translational processing.

The other DUB displaying a large activity towards polyUbs is Otulin, an enzyme recently identified as a regulator of signaling cascades controlled by the ubiquitin-ligase linear-ubiquitin-assembly complex (LUBAC)^44^,^45^. Otulin cleaves in random manner both anchored and unanchored linear ubiquitin chains and thus, unlike USP5, it should be able to process ribosome-associated nascent polyUbs, provided that they contain two adjacent folded ubiquitin moieties^44^. Although no attempts were made to confirm the participation of Otulin in co-translational processing of polyUbs (the anti-Otulin antibody does not work in immunodepletion experiments) our data suggest a role for Otulin at this level because, to the best of our knowledge, there is no other mammalian DUB with the capacity to cleave linear ubiquitin chains that is insensitive to HA-UbVME but sensitive to HA-Ubal. Thus, regardless of the pathway that prevails *in vivo*, if any, co- or post-translational, our data suggest that Otulin is a major player in polyUb processing. It is tempting to speculate that this function of Otulin aims at avoiding any interference between polyUb synthesis and the signaling cascades referred above.

An interesting aspect that emerges from our work is that every peptide bond that has to be hydrolyzed during processing of each ubiquitin precursor can be efficiently cleaved by at least two different DUBs. In the case of Ub-RPs, where a single hydrolysis event yields free ubiquitin, UCHL3 (in both mouse liver and HeLa cells), USP9X (in mouse liver) and USP7 (in HeLa cells) are the main enzymes in charge. For polyUbs, several processing pathways can be inferred. These include: 1) co- or post-translational cleavage of ubiquitin-ubiquitin peptide bonds by Otulin complemented by the action of UCHL3 to remove the C-terminal extension, and 2) post-translational processing by USP5 preceded or not by the action of UCHL3. The redundancy of this enzymatic system provides a rationale to understand the robustness of the ubiquitin *de novo* synthesis pathway.

Understanding the function of the many DUBs present in any eukaryotic organism remains a major challenge. The redundancy of these enzymes^56^ and their substrate promiscuity^57^,^58^ can raise difficulties in any experimental approach aiming at allocating DUBs to particular substrates or biological pathways. The strategy employed in this work, although relying on *in vitro* assays which will have to be validated by *in vivo* studies, is relatively insensitive to the redundancy problem. It also presents the advantage of identifying DUBs taking into consideration not only their substrate specificities and catalytic efficiencies but also their relative endogenous abundances. As such, it provides an alternative and informative view on the biological roles of this complex family of enzymes.

## Methods

### DNA constructs and recombinant proteins

Oligonucleotides used in DNA manipulations are listed in Supplementary Table S2. cDNAs encoding mouse UBB and UBC, and human UBB were obtained by PCR of genomic DNA. Mouse cDNAs were first cloned into HindIII/BamHI-digested pGEM4 (Promega) and the plasmids were subjected to mutagenesis, using the QuikChange® Site-Directed Mutagenesis kit (Agilent), to introduce an NdeI site at their start ATG codon. These cDNAs and the one encoding human UBB were digested with NdeI and BamHI and cloned into pET23a (Novagen). A cDNA encoding UBA52 (the mouse and human proteins are identical) was obtained by PCR using human fibroblasts cDNA. The cDNA encoding human UBA80 was PCR amplified from RPS27A-pBluescriptR (PlasmID repository; clone ID HsCD00327233). These cDNAs were digested with NdeI/BamHI and cloned into pET23a (Novagen). Mouse UBA80 in pET23a was generated from the human construct by changing alanine 133 codon to GGA (glycine). The same strategy was used to obtain the plasmid encoding mouse UBBY305P. cDNAs encoding UBA52, UBA80 and UBC lacking a stop codon were obtained by PCR using the pET23a-based plasmids. For mouse UBB a different strategy was used. The two last codons of UBB (TATTAA; encoding the last tyrosine and the stop codon) were replaced by TACGTA. The plasmid was then digested with SnaBI which cuts bluntly after the new Y305 codon. Untagged ubiquitin precursors were expressed in BL21 (DE3) or Rosetta2 (DE3) *E. coli* strains by induction with 0.5 mM IPTG for 2–3 h at 37 °C. Cells were lysed by sonication and subjected to a clarifying spin (17,000*g*, 15 min, 4 °C). To purify UBB proteins, cell lysates prepared in 50 mM Tris-HCl, pH 8.5, 1 mM EDTA-NAOH, pH 8.0, 0.5 mM DTT, 1:500 (v/v) mammalian protease inhibitor cocktail (Sigma), 0.25 mg/ml PMSF were applied to a HiTrap Q HP 1-ml anion exchange column (GE Healthcare) and the proteins were collected in the flow-through. Mouse UBC presented some degradation products. To enrich for full-length protein, we explored the presence of two cysteine residues at its C terminus. Briefly, cell lysates prepared in buffer A (20 mM HEPES, pH 7.2, 150 mM NaCl, 1 mM EDTA-NAOH, pH 8.0, 0.2% (w/v) Triton X-100) supplemented with 0.2 mM DTT and protease inhibitors (see above), were incubated with Thiopropyl Sepharose 6B (GE Healthcare) for 2 h at 4 °C. The beads were washed twice with the buffer A, twice with a high salt buffer (20 mM HEPES, pH 7.2, 0.5 M NaCl, 1 mM EDTA-NaOH, pH 8.0) and twice with a low salt buffer (5 mM HEPES, pH 7.2, 50 mM NaCl, 1 mM EDTA-NaOH, pH 8.0). UBC was eluted with the low salt buffer containing 50 mM DTT (1 h, 4 °C), and dialyzed against 20 mM HEPES, pH 7.2, 50 mM NaCl, and 1 mM DTT. Note that UBC is prone to aggregation. Cell lysates containing UBA52 and UBA80 were subjected to chromatography using a HiTrap SP HP 1-ml column (GE Healthcare) and a 20-ml linear gradient of 0.1–1 M NaCl in 20 mM HEPES, pH 7.2, 0.5 mM DTT. The cDNAs encoding human UCHL3 and Otulin were amplified from UCHL3-pCMV-Sport6 (PlasmID repository; clone ID HsCD00327233) and Fam105b-pCMV-Sport6 (PlasmID repository; clone ID HsCD00331143) and inserted into the NdeI/XhoI and NdeI/BamHI sites of pET28a (Novagen), respectively. Expression plasmids encoding His_6_-tagged human wild type USP5 (Addgene plasmid 25299) and USP5(C335A) were kindly provided by Dr. Cheryl Arrowsmith (University of Toronto, Canada). His_6_-tagged USP5 proteins were purified using Ni-Sepharose 6 Fast Flow beads (GE Healthcare) as described^51^. Otulin and UCHL3 in 50 mM Tris-HCl, pH 8.0, 150 mM NaCl, 10 mM imidazole, 10% (w/v) glycerol, 1 mM DTT were purified likewise. Recombinant proteins were stored in 50 mM Tris-HCl, pH 8.0, 150 mM NaCl, 10% (w/v) glycerol, 1 mM DTT at −80 °C. HA-tagged ubiquitin, HA-Ubal and HA-UbVME were produced as described^39^.

### *In vitro* synthesis of proteins

Radiolabeled proteins were synthesized using the TNT® T7 quick coupled transcription/translation kit (Promega) in the presence of [^35^S]methionine (specific activity >1000 Ci/mmol; PerkinElmer Life Sciences). Where indicated DUB inhibitors (20 μM, final concentration), puromycin (3 mM; Sigma) and cycloheximide (2 mM; Sigma) were used. After SDS-PAGE, proteins were blotted onto a nitrocellulose membrane and subjected to autoradiography.

### Cell culture and siRNA transfections

HeLa cells were grown at 37 °C in the presence of 5% CO_2_ in standard DMEM supplemented with 10% (v/v) of heat-inactivated FBS, 100 U/ml penicillin, 100 μg/ml streptomycin, 1% (v/v) fungizone and 1% (v/v) kanamycin (Invitrogen). Cells were transfected with siRNAs (10 nM, final concentration) using Lipofectamine RNAiMAX (Invitrogen) according to the manufacturer and harvested 3 days later. Custom siRNA oligos against Otulin (#1: GACUGAAAUUUGAUGGGAAtt; #2: CAAAUGAGGCGGAGGAAUAtt; Sigma) and UCHL3 (#1: GGCACCAAGUAUAGAUGAGtt; #2 GGGACAAGAUGUUACAUCAtt; Sigma) have been described^44^,^59^. A 1:1 mixture of the two oligos was used. ON-TARGETplus Non-targeting siRNA #1 (D-001810-01-05; ThermoScientific) served as a negative control. Cells were harvested with StemPro® Accutase® Cell Dissociation Reagent (Gibco), washed with ice-cold PBS, and collected by centrifugation at 4 °C.

### Subcellular fractionation and SEC

HeLa cell total extracts were obtained by sonication in SEM buffer (0.25 M sucrose, 20 mM MOPS-KOH, pH 7.4, 1 mM EDTA-NaOH, pH 8.0, 1 mM DTT) containing 0.1% (w/v) Triton X-100, 0.1 mg/ml PMSF and 1:500 (v/v) mammalian protease inhibitor cocktail (Sigma). For the preparation of cytosol, cell pellets were resuspended in five volumes of buffer B (20 mM Tris-HCl, pH 7.5, 150 mM NaCl, 1 mM EDTA-NaOH, pH 8.0, 10% (w/v) glycerol) supplemented with 1 mM DTT, 1:500 (v/v) mammalian protease inhibitor cocktail (Sigma) and 0.1 mg/ml PMSF, lysed using a Dounce homogenizer, and centrifuged at 17,000 *g* for 15 min at 4 °C. The supernatant was further centrifuged for 30 min at 100,000 *g* at 4 °C to obtain the cytosolic fraction. Mouse liver cytosol was prepared exactly as described^39^. Cytosolic proteins (6–8 mg in 200 μl) were injected into a Superose 12 10/300 GL column (GE Healthcare) running in buffer B supplemented with 0.5 mM DTT at a flow rate of 0.5 ml/min; 0.5-ml fractions were collected. The protein standards were: thyroglobulin (670 kDa), ferritin (440 kDa), alcohol dehydrogenase (150 kDa), bovine serum albumin (66 kDa), ovalbumin (45 kDa) and soybean trypsin inhibitor (21.5 kDa). The C57BL/6J mice and Wistar rats were handled in accordance with the protocols approved by the IBMC Animal Ethics Committee (CEA—Comissão de Ética Animal). Experimental protocols were approved by the Portuguese General Veterinarian Board (DGAV – Direcção Geral de Alimentação e Veterinária).

### Activity assays

Cytosolic proteins (100 μg) or aliquots from SEC fractions (equivalent to 1.2 mg of total cytosolic proteins) were reacted with 1 μM HA-UbVME in 100–120 μl of buffer B, 1 mM DTT, for 20 min at 37 °C. Deubiquitination assays were done in 20 μl of buffer B, 1 mM DTT for 30 min at 37 °C. Reactions contained 3–4 μg of ubiquitin precursors and cytosolic proteins (25–50 μg) or SEC fractions (from 100–200 μg of cytosolic proteins). HA-UbVME or HA-Ubal (20 μM, final concentration) were incubated with protein fractions for 15 min at 37 °C before adding the substrates. Digestions with His_6_-UCHL3 (200 ng) were done for 30 min at 37 °C. Thioester assays were performed exactly as described^60^.

### Pegylation assays

Samples in buffer B, 1 mM Tris(2-carboxyethyl)phosphine hydrochloride (TCEP) were supplemented with SDS (2%, final concentration), incubated for 10 min at 60 °C, and treated with 5 mM methoxypolyethylene glycol maleimide 5,000 (added from a 20 mM stock solution in 50 mM Tris-HCl, pH 7.5; Fluka/Sigma-Aldrich) for 1 h at 25 °C. After adding 0.2 M DTT (final concentration), samples were precipitated with 10% (w/v) trichloroacetic acid (final concentration), and processed for SDS-PAGE.

### Antibodies and immunoprecipitations

Antibodies against human USP4 (A300-830A), USP5 (A301-542A), USP7 (A300-033A), USP8 (A302-929A), USP9X (A301–351A), USP15 (A300-923A), USP48 (A301-190A) and UCHL3 (A302-948A) were from Bethyl Laboratories. A second anti-UCHL3 antibody was from Santa Cruz (sc-23855). The anti-Otulin antibody was from Atlas Antibodies (HPA051074). Anti-Otulin antibodies AP5734c (Abgent) and sc-162789 (Santa Cruz) did not provide satisfactory results. Anti-HA (16B12) was from Covance. Rabbit IgGs were purified using protein A-Sepharose beads (Sigma). Immunoprecipitation/immunodepletion assays and affinity purification of HA-UbVME-labeled proteins were done as described^39^.

### Protein identification by nano-HPLC-MALDI-TOF/TOF

Protein bands from SDS-gels were digested with trypsin (Promega V5111) and the peptides were separated by nano-HPLC using standard conditions^60^. The eluted peptides were mixed with a continuous flow of α-CHCA matrix solution and applied directly on a MALDI plate using a fraction collector Probot (Dionex, Amsterdam). Spectra were obtained on a MALDI TOF/TOF mass spectrometer (4800 Proteomics Analyzer, Applied Biosystems, Foster City, CA, USA) in the positive ion reflector mode. 16 of most intense peaks in each sample spot were selected for subsequent MS/MS (2kV). Spectra were analyzed with T2S (v1.0, Matrix Science Ltd, U.K) and submitted in Mascot software (v.2.1.0.4, Matrix Science Ltd, U.K.) for protein/peptide identification based on MS/MS data using the following criteria: trypsin as enzyme; a maximum of two missed cleavages; mass tolerances of 20 ppm for peptide precursors, mass tolerance of 0.3 Da for fragment ions; database SwissProt (*Homo sapiens*, release date 01052013). Protein identifications based on MS/MS data were considered reliable when the Mascot ion score confidence level for each individual peptide was higher than 32.

## Additional Information

**How to cite this article**: Grou, C. P. *et al.* The *de novo* synthesis of ubiquitin: identification of deubiquitinases acting on ubiquitin precursors. *Sci. Rep.* **5**, 12836; doi: 10.1038/srep12836 (2015).

## Supplementary Material

Supplementary Information
