# Supplementary material for: The de novo synthesis of ubiquitin: identification of deubiquitinases acting on ubiquitin precursors
Source: Sci Rep. 2015 Aug 3;5:12836. doi: 10.1038/srep12836 (PMC4522658; doi:10.1038/srep12836)
Supplement: Supplementary Information [file srep12836-s1.pdf]

Supplementary Information for

The *de novo* synthesis of ubiquitin: identification of deubiquitinases acting on ubiquitin precursors

Cláudia P. Grou[1,2,#], Manuel P. Pinto[1,2,#], Andreia V. Mendes[1,2], Pedro Domingues[3], and Jorge E. Azevedo[1,4,*]

[1] Instituto de Investigação e Inovação em Saúde, Universidade do Porto, Portugal

[2] Organelle Biogenesis and Function Group, Instituto de Biologia Molecular e Celular (IBMC), Universidade do Porto, Rua do Campo Alegre 823, 4150-180 Porto, Portugal

[3] Mass Spectrometry Centre, UI-QOPNA, Department of Chemistry, University of Aveiro, 3810-193 Aveiro, Portugal

[4] Instituto de Ciências Biomédicas Abel Salazar (ICBAS), Universidade do Porto, Rua de Jorge Viterbo Ferreira 228, 4050-313 Porto, Portugal

[#] These authors contributed equally to this work.

*Corresponding author:

Jorge E. Azevedo

e-mail: jazevedo@ibmc.up.pt

Supplementary Fig. S1

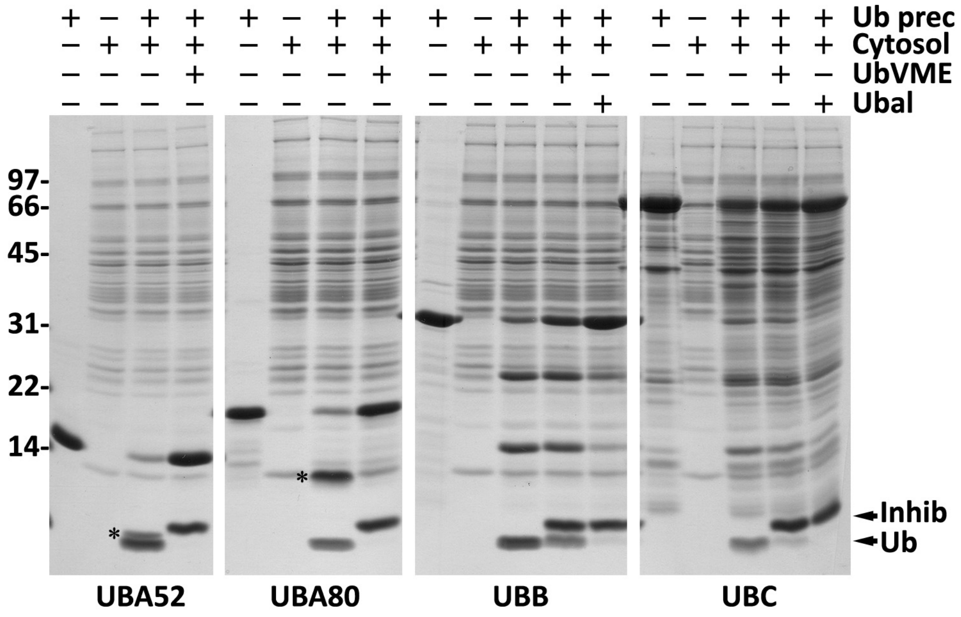

Supplementary Figure S1- Processing of recombinant mouse ubiquitin precursors by a mouse liver cytosolic fraction. Mouse ubiquitin precursors (Ub prec) were incubated with mouse liver cytosolic proteins in the absence or presence of HA-UbVME or HA-Ubal, as indicated. The samples were analyzed by SDS-PAGE and Coomassie blue-stained gels are presented. The asterisks indicate ribosomal proteins L40 and S27A released by processing of UBA52 and UBA80, respectively. Numbers to the left indicate the molecular weights of protein standards in kDa. Inhib, HA-UbVME or HA-Ubal. Ub, ubiquitin.

Supplementary Fig. S2

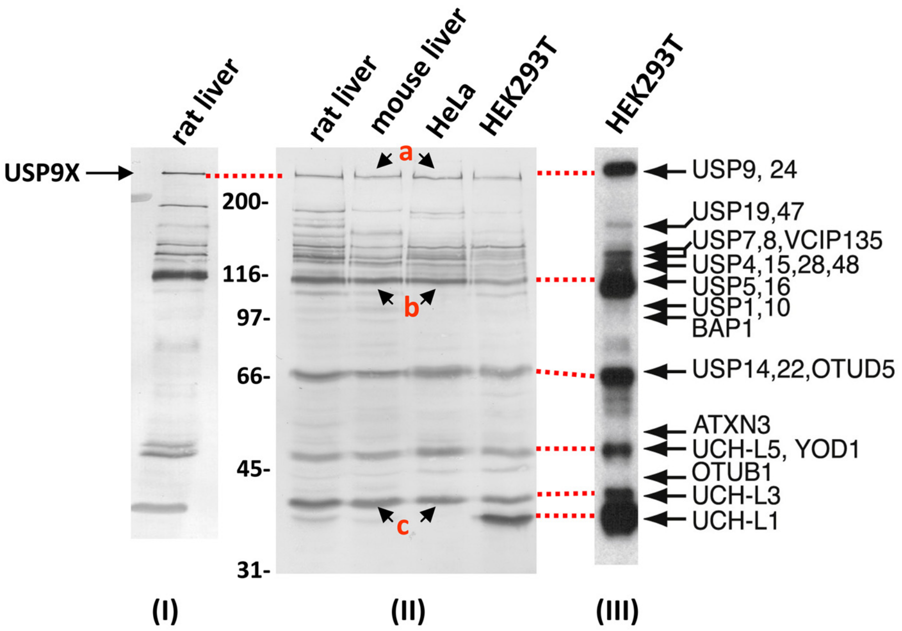

Supplementary Figure S2- SDS-PAGE profiles of HA-UbVME-reactive protein bands from rat and mouse liver cytosol and HeLa and HEK293T cells. The HA-UbVME labeling pattern obtained for the indicated extracts (panel II) is compared with previously published ones (panels I and III). Panel I is from Fig. 2 in Grou C. P. *et al.* (2012) *J. Biol. Chem.* 287, 12815-12827 (© the American Society for Biochemistry and Molecular Biology). Panel III is from Fig. 5 in Altun M. *et al.* (2011) *Chem. Biol.* 18, 1401-1412 (with permission from Elsevier). Indicated DUBs were identified by MS in the above mentioned studies. Protein bands a, b and c refer to HA-UbVME-reactive bands that co-elute with the enzymatic activities described in this work.

Supplementary Fig. S3

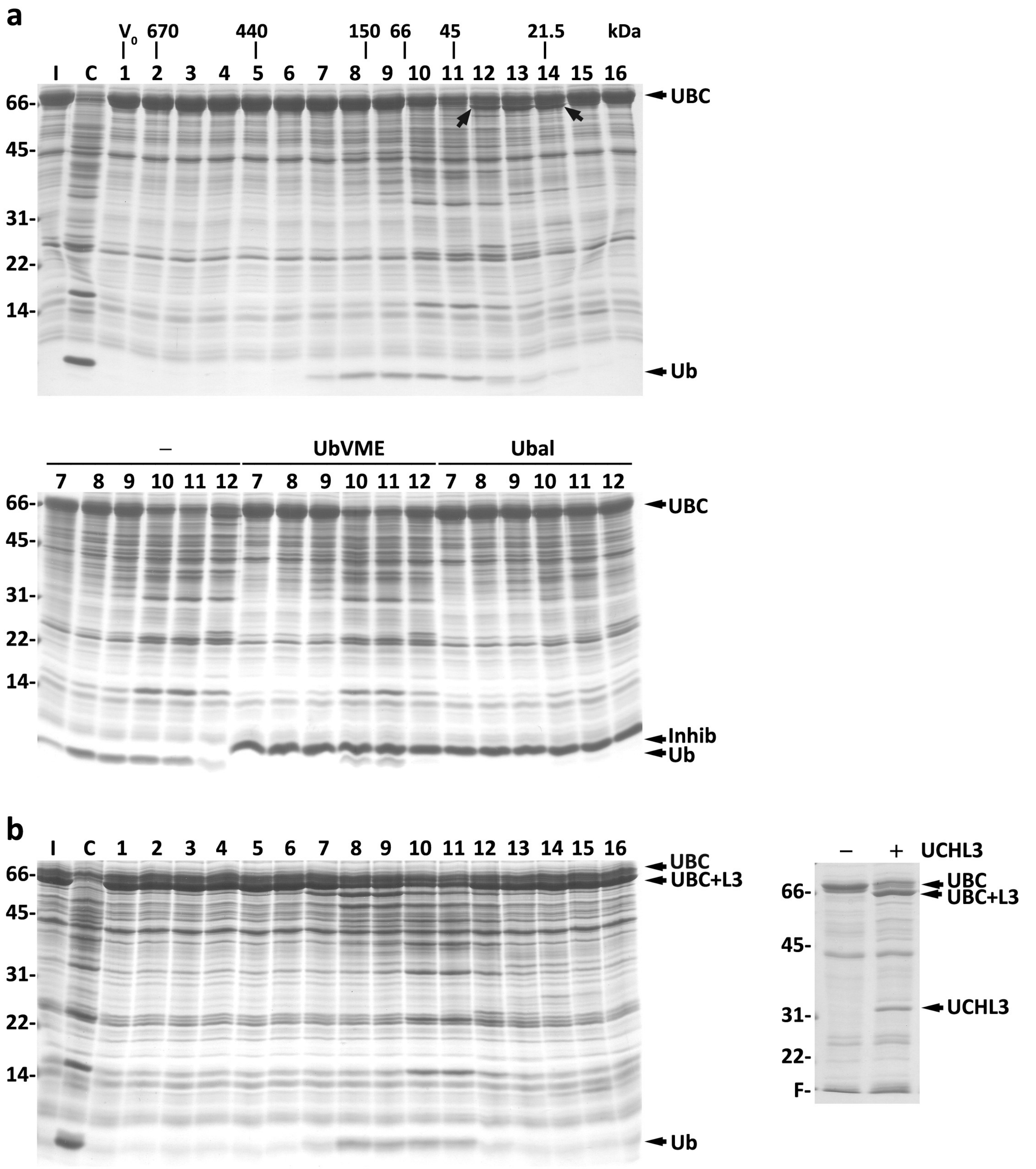

Supplementary Figure S3- Characterization of mouse liver cytosolic DUBs acting on UBC. (**a**) SDS-PAGE/Coomassie blue staining analysis of UBC processing by mouse liver cytosolic SEC fractions (lanes 1-16). Lane C, total cytosolic proteins were also assayed (upper panel). The arrows indicate a cleavage product of UBC most probably reflecting removal of its 50-amino acid residues C-terminal extension by UCHL3, which is present in these fractions. Protein standards used to calibrate the column, as well as the void volume (V_0), are indicated. SEC fractions 7-12 were preincubated in the absence or presence of HA-UbVME or HA-Ubal and used in UBC processing activity assays (lower panel). Inhib, HA-UbVME or HA-Ubal. (**b**) Left panel, exactly as in (**a**), but using UCHL3-pretreated UBC. Note that a fraction of UBC is not cleaved by UCHL3, probably because some UBC forms aggregates. Lanes I, recombinant UBC proteins used in the assays. Right panel, SDS-PAGE analysis of UBC incubated in the absence or presence of UCHL3. Ubiquitin (Ub), UBC, UBC lacking the C-terminal extension (UBC+L3) and recombinant UCHL3 are indicated. F, front of the gel. Numbers to the left indicate the molecular weights of protein standards in kDa.

Supplementary Fig. S4

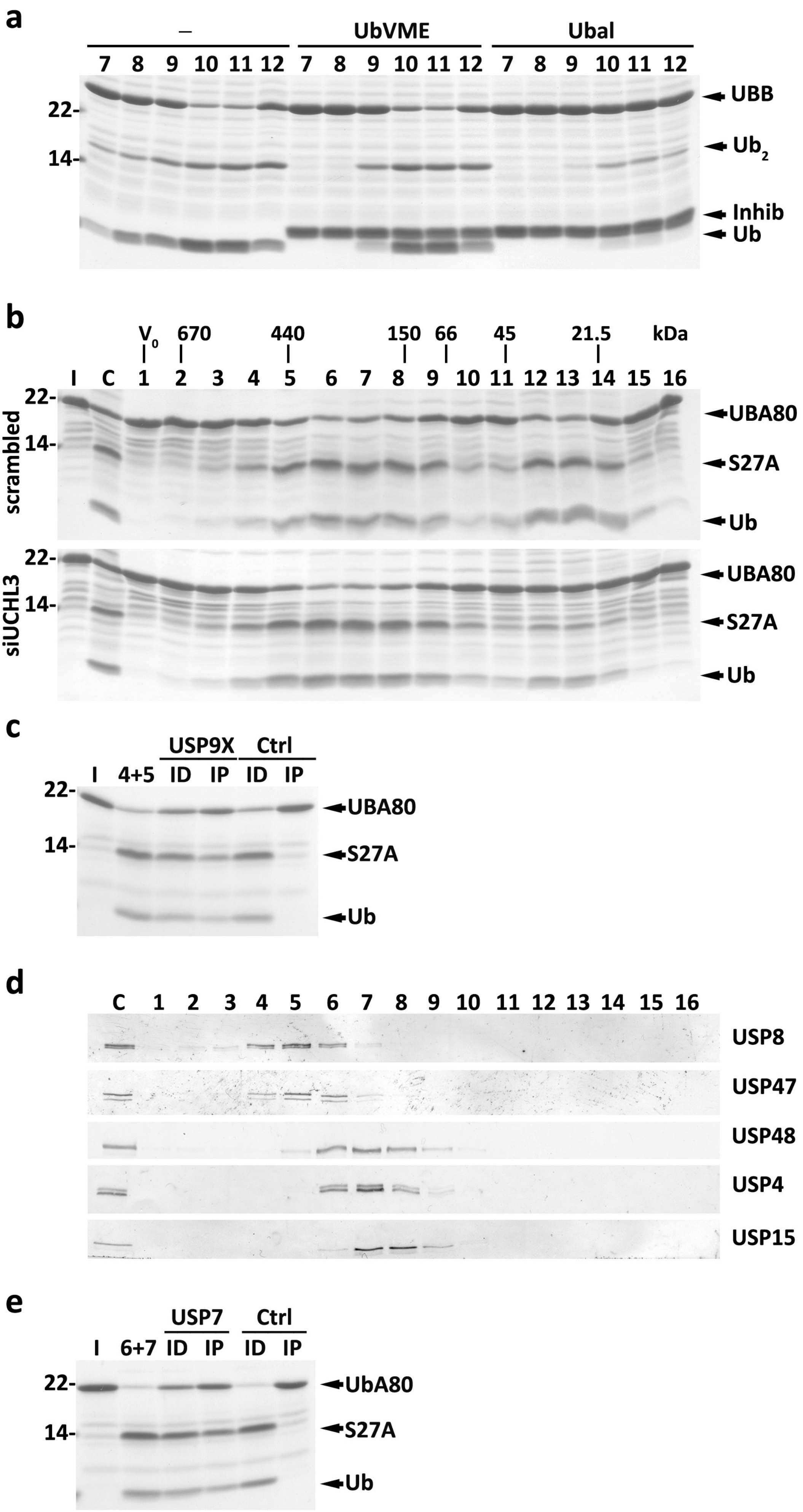

Supplementary Figure S4- Characterization of HeLa cells cytosolic DUBs acting on ubiquitin precursors. (**a**) The UBB cleavage SEC activity peak comprises an HA-UbVME-sensitive and an HA-UbVME-insensitive component. Fractions 7-12 from SEC were preincubated in the absence or presence of HA-UbVME or HA-Ubal and used in UBB processing activity assays. Inhib, HA-UbVME or HA-Ubal. UBB, the cleavage intermediate (Ub_2) and ubiquitin (Ub) are indicated. (**b**) SEC profiles of UBA80-cleavage activity in control (scrambled) and *UCHL3* knocked-down (siUCHL3) HeLa cells cytosol. Lane C, total cytosolic proteins from both control and *UCHL3* knocked-down cells were also assayed. Protein standards used to calibrate the column, as well as the void volume (V_0), are indicated. (**c**) Pooled SEC fractions 4 and 5 were subjected to an immunoprecipitation/immunodepletion assay using control (lanes Ctrl) or anti-USP9X IgGs (lanes USP9X). Pooled fractions (4+5) and the corresponding immunoprecipitated (lanes IP) and immunodepleted fractions (lanes ID) were assayed for UBA80 cleavage. (**d**) SEC elution profiles of HeLa DUBs identified by mass spectrometry analysis of protein bands indicated in Fig. 6e. (**e**) Immunoprecipitation/immunodepletion assay exactly as described in (**c**), but using pooled SEC fractions 6 and 7, and anti-USP7 IgGs. UBA80 and its cleavage products, ribosomal protein S27A and ubiquitin (Ub), are indicated. Lanes I, recombinant UBA80 used in the assays. Numbers to the left indicate the molecular weights of protein standards in kDa.

Supplementary Fig. S5

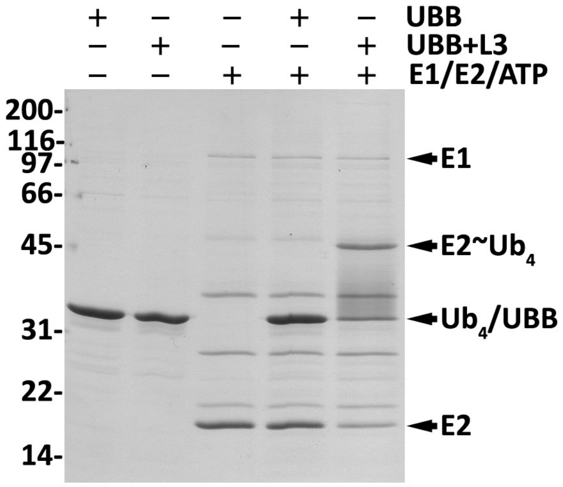

Supplementary Fig. S5- Thioester assay showing that recombinant mouse UBB contains its C-terminal Tyr residue. Recombinant mouse UBB pretreated (lanes UBB+L3) or not (lanes UBB) with UCHL3 was incubated with E1, the E2 UbcH7 and inorganic pyrophosphatase in buffer B supplemented with ATP and $MgCl_2$ for 15 min at 37 ºC. The assay was stopped with N-ethylmaleimide. Samples were analyzed by SDS-PAGE under nonreducing conditions. A Coomassie blue-stained gel is shown. Note that only UCHL3-treated recombinant mouse UBB can be activated by the E1 and transferred to UbcH7 yielding the corresponding thioester (E2~Ub_4), showing that the untreated UBB contains its C-terminal Tyr residue. Numbers to the left indicate the molecular weights of protein standards in kDa.

Supplementary Fig. S6

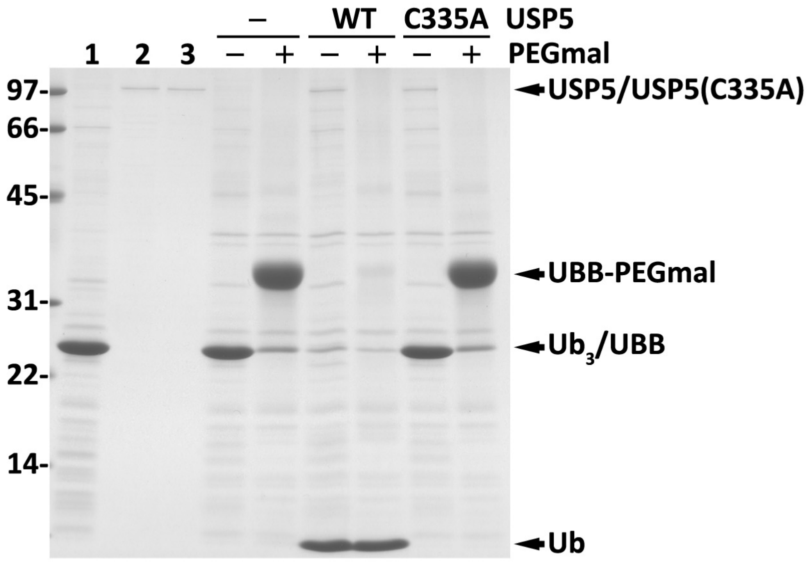

Supplementary Fig. S6- Control assay showing that removal of the C-terminal cysteine residue from human UBB is dependent on the catalytic activity of USP5. Recombinant human UBB was incubated in the absence or presence of 50 ng of recombinant USP5 wild type (WT) or the catalytically inactive USP5(C335A), as indicated, for 30 min at 37 ºC. Samples were then subjected to pegylation (+ PEGmal) and analyzed by SDS-PAGE. A Coomassie blue-stained gel is shown. Lanes 1, 2 and 3, recombinant human UBB, USP5 wild type and USP5(C335A), respectively, used in the assay. UBB-PEGmal, pegylated UBB species. Numbers to the left indicate the molecular weights of protein standards in kDa.

Supplementary Table S1- Proteins identified by MS in bands 1-6 from the gel in Fig. 6e

Band	Protein	Score	Mass	Matches[a]	Sequences[b]	Description
1	UBP47_HUMAN	580	157212	12(12)	12(12)	Ubiquitin carboxyl-terminal hydrolase 47 OS=Homo sapiens GN=USP47 PE=1 SV=3
	PPT1_HUMAN	148	34171	2(2)	1(1)	Palmitoyl-protein thioesterase 1 OS=Homo sapiens GN=PPT1 PE=1 SV=1
	S10AB_HUMAN	139	11733	1(1)	1(1)	Protein S100-A11 OS=Homo sapiens GN=S100A11 PE=1 SV=2
	ECH1_HUMAN	58	35793	1(1)	1(1)	Delta(3,5)-Delta(2,4)-dienoyl-CoA isomerase, mitochondrial OS=Homo sapiens GN=ECH1 PE=1 SV=2
	RS27A_HUMAN	49	17953	1(1)	1 (1)	Ubiquitin-40S ribosomal protein S27a OS=Homo sapiens GN=RPS27A PE=1 SV=2
	TMPSD_HUMAN	28	62659	1(1)	1(1)	Transmembrane protease serine 13 OS=Homo sapiens GN=TMPRSS13 PE=2 SV=3
	OXR1_HUMAN	22	97909	2(1)	1(1)	Oxidation resistance protein 1 OS=Homo sapiens GN=OXR1 PE=1 SV=2
2	S10AB_HUMAN	124	11733	1(1)	1(1)	Protein S100-A11 OS=Homo sapiens GN=S100A11 PE=1 SV=2
	RSP27A_HUMAN	46	17953	1(1)	1(1)	Ubiquitin-40S ribosomal protein S27a OS=Homo sapiens GN=RPS27A PE=1 SV=2
	PPT1_HUMAN	44	34171	1(1)	1(1)	Palmitoyl-protein thioesterase 1 OS=Homo sapiens GN=PPT1 PE=1 SV=1
	UBP8_HUMAN	34	127444	1(1)	1(1)	Ubiquitin carboxyl-terminal hydrolase 8 OS=Homo sapiens GN=USP8 PE=1 SV=1
	ASAP2_HUMAN	31	111581	1(1)	1(1)	Arf-GAP with SH3 domain, ANK repeat and PH domain-containing protein 2 OS=Homo sapiens GN=ASAP2 PE=1 SV=3
	S6OS1_HUMAN	31	68123	1(1)	1(1)	Protein SIX6OS1 OS=Homo sapiens GN=SIX6OS1 PE=2 SV=2
	NDC80_HUMAN	17	73867	1(1)	1(1)	Kinetochore protein NDC80 homolog OS=Homo sapiens GN=NDC80 PE=1 SV=1
3	UBP7_HUMAN	888	128220	24(22)	24(22)	Ubiquitin carboxyl-terminal hydrolase 7 OS=Homo sapiens GN=USP7 PE=1 SV=2
	RS27A_HUMAN	190	17953	4(4)	4(4)	Ubiquitin-40S ribosomal protein S27a OS=Homo sapiens GN=RPS27A PE=1 SV=2
	S10AB_HUMAN	98	11733	1(1)	1(1)	Protein S100-A11 OS=Homo sapiens GN=S100A11 PE=1 SV=2
	UBP48_HUMAN	76	118956	1(1)	1(1)	Ubiquitin carboxyl-terminal hydrolase 48 OS=Homo sapiens GN=USP48 PE=1 SV=1
	UBP8_HUMAN	74	127444	2(2)	2(2)	Ubiquitin carboxyl-terminal hydrolase 8 OS=Homo sapiens GN=USP8 PE=1 SV=1
	FAKD2_HUMAN	31	81410	1(1)	1(1)	FAST kinase domain-containing protein 2 OS=Homo sapiens GN=FASTKD2 PE=1 SV=1
	TF3B_HUMAN	30	73794	1(1)	1(1)	Transcription factor IIIB 90 kDa subunit OS=Homo sapiens GN=BRF1 PE=1 SV=1
	FAAA_HUMAN	29	46344	1(1)	1(1)	Fumarylacetoacetase OS=Homo sapiens GN=FAH PE=1 SV=2
	LBN_HUMAN	24	147856	2(1)	2(1)	Limbin OS=Homo sapiens GN=EVC2 PE=1 SV=1
	UBP15_HUMAN	19	112348	1(1)	1(1)	Ubiquitin carboxyl-terminal hydrolase 15 OS=Homo sapiens GN=USP15 PE=1 SV=3
4	UBP7_HUMAN	254	128220	6(6)	6(6)	Ubiquitin carboxyl-terminal hydrolase 7 OS=Homo sapiens GN=USP7 PE=1 SV=2
	UBP48_HUMAN	221	118956	4(4)	4(4)	Ubiquitin carboxyl-terminal hydrolase 48 OS=Homo sapiens GN=USP48 PE=1 SV=1
	S10AB_HUMAN	120	11733	1(1)	1(1)	Protein S100-A11 OS=Homo sapiens GN=S100A11 PE=1 SV=2
	RS27A_HUMAN	57	17953	1(1)	1(1)	Ubiquitin-40S ribosomal protein S27a OS=Homo sapiens GN=RPS27A PE=1 SV=2
	TMPSD_HUMAN	20	62659	1(0)	1(0)	Transmembrane protease serine 13 OS=Homo sapiens GN=TMPRSS13 PE=2 SV=3
5	UBP15_HUMAN	620	112348	18(18)	18(18)	Ubiquitin carboxyl-terminal hydrolase 15 OS=Homo sapiens GN=USP15 PE=1 SV=3
	UBP48_HUMAN	232	118956	7(7)	7(7)	Ubiquitin carboxyl-terminal hydrolase 48 OS=Homo sapiens GN=USP48 PE=1 SV=1
	S10AB_HUMAN	145	11733	2(2)	1(1)	Protein S100-A11 OS=Homo sapiens GN=S100A11 PE=1 SV=2
	RS27A_HUMAN	130	17953	5(5)	4(4)	Ubiquitin-40S ribosomal protein S27a OS=Homo sapiens GN=RPS27A PE=1 SV=2
	UBP5_HUMAN	72	95725	2(2)	2(2)	Ubiquitin carboxyl-terminal hydrolase 5 OS=Homo sapiens GN=USP5 PE=1 SV=2
	TMPSD_HUMAN	35	62659	1(1)	1(1)	Transmembrane protease serine 13 OS=Homo sapiens GN=TMPRSS13 PE=2 SV=3
	UBP7_HUMAN	28	128220	1(1)	1(1)	Ubiquitin carboxyl-terminal hydrolase 7 OS=Homo sapiens GN=USP7 PE=1 SV=2
	SMO_HUMAN	23	86341	1(1)	1(1)	Smoothened homolog OS=Homo sapiens GN=SMO PE=1 SV=1
6	UBP4_HUMAN	353	108496	12(11)	12(11)	Ubiquitin carboxyl-terminal hydrolase 4 OS=Homo sapiens GN=USP4 PE=1 SV=3
	UBP15_HUMAN	270	112348	9(9)	9(9)	Ubiquitin carboxyl-terminal hydrolase 15 OS=Homo sapiens GN=USP15 PE=1 SV=3
	S10AB_HUMAN	109	11733	1(1)	1(1)	Protein S100-A11 OS=Homo sapiens GN=S100A11 PE=1 SV=2
	RS27A_HUMAN	98	17953	3(3)	3(3)	Ubiquitin-40S ribosomal protein S27a OS=Homo sapiens GN=RPS27A PE=1 SV=2
	UBP5_HUMAN	25	95725	1(1)	1(1)	Ubiquitin carboxyl-terminal hydrolase 5 OS=Homo sapiens GN=USP5 PE=1 SV=2

[a] Count of MS/MS spectra that have been matched to peptides from this protein. Number in brackets refer to the count of significant matches.

[b] Count of matches to distinct peptide sequences. Number in brackets refer to the count of sequences with significant matches.

Supplementary Table S2- Primers used in cloning and mutagenesis procedures

Plasmid/PCR product	Primers
MmUBB-pGEM4	Forward: 5'-GCGCGAAGCTTAGGTCAAAATGCAGATCTT-3'<br>Reverse: 5'-GGGGGGATCCGCAGACCGAATAATTAATAGC-3'
MmUBC-pGEM4	Forward: 5'-GCGCGAAGCTTATTTTGTGACAGACGATGCAG-3'<br>Reverse: 5'-GCTAGGATCCACACCTCCCCCATCACAC-3'
MmUBB(NdeI)-pGEM4	Forward: 5'-CCAAGCTTAGGTCCATATGCAGATC-3'<br>Reverse: 5'-GATCTGCATATGGACCTAAGCTTGG-3'
MmUBC(NdeI)-pGEM4	Forward: 5'-CTTATTTTGTGACAGCATATGCAGATC-3'<br>Reverse: 5'-GATCTGCATATGCTGTCACAAAATAAG-3'
MmUBBY305P-pET23a	Forward: 5'-TCGACCCTGCACCTGGTCCTCCGTCTGAGGGGTGGTCCTTAAGGTACCGC-3<br>Reverse: 5'-GGCCGCGGTACCTTAAGGACCACCCCTCAGACGGAGGACCAGGTGCAGGG-3'
HsUBB-pET23a	Forward: 5'-TTAAACTAGGTCCATATGCAGATCTT-3'<br>Reverse: 5'-GCGGGATCCTGCCATGACTGAAGAATTAAC-3'
HsUBA52-pET23a	Forward: 5'-GCGGCGCCCATATGCAGATCTTTGTGAAGAC-3'<br>Reverse: 5'-GCGCGCGGATCCTTATTTGACCTTCTTCTTG-3'
HsUBA80-pET23a	Forward: 5'-GCGGCGCCCATATGCAGATTTTCGTGAAAAC-3'<br>Reverse: 5'-GCGCGGATCCTTACTTGTCTTCTGGTTTGTTG-3'
MmUBA80-pET23a	Forward: 5'-GCTGGGGTGTTTATGGGAAGTCACTTTGACAG-3'<br>Reverse: 5'-CTGTCAAAGTGACTTCCCATAAACACCCCAGC-3'
HsUCHL3-pET28a	Forward: 5'-GGCTAATGCATATGGAGGGTCAACGCTGG-3'<br>Reverse: 5'-GCCGCCAACTCGAGCTATGCTGCAGAAAGAGC-3'
HsOtulin-pET28a	Forward: 5'-GAGTCGGCCATATGAGTCGGGGGACTATGC-3'<br>Reverse: 5'-GCCGCGGATCCTCATAGACTGGTCTCCTCACACAC-3'
MmUBB(SnaBI)-pET23a	Forward: 5'-GGTGGCTACGTATTATTCGGTCTG-3'<br>Reverse: 5'-CAGACCGAATAATACGTAGCCACC-3'
MmUBA52ΔSTOP	Forward: 5'-CCGCGAAATTAATACGAC-3'<br>Reverse: 5'-TTTGACCTTCTTCTTGGGAC-3'
MmUBA80ΔSTOP	Forward: 5'-CCGCGAAATTAATACGAC-3'<br>Reverse: 5'-CTTGTCTTCTGGTTTGTTG-3'
MmUBCΔSTOP	Forward: 5'-CCGCGAAATTAATACGAC-3'<br>Reverse: 5'-CACCCAAGAACAAGCACAAG-3'
